# Database of exact tandem repeats in the Zebrafish genome

**DOI:** 10.1186/1471-2164-11-347

**Published:** 2010-06-01

**Authors:** Eric C Rouchka

**Affiliations:** 1 Department of Computer Engineering and Computer Science, Speed School of Engineering, University of Louisville, Duthie Center, Room 208, Louisville, KY USA

## Abstract

**Background:**

Sequencing of the approximately 1.7 billion bases of the zebrafish genome is currently underway. To date, few high resolution genetic maps exist for the zebrafish genome, based mainly on single nucleotide polymorphisms (SNPs) and short microsatellite repeats. The desire to construct a higher resolution genetic map led to the construction of a database of tandemly repeating elements within the zebrafish Zv8 assembly.

**Description:**

Exact tandem repeats with a repeat length of at least three bases and a copy number of at least 10 were reported. Repeats with a total length of 250 or fewer bases and their flanking regions were masked for known vertebrate repeats. Optimal primer pairs were computationally designed in the regions flanking the detected repeats. This database of exact tandem repeats can then be used as a resource by molecular biologists with interests in experimentally testing VNTRs within a zebrafish population.

**Conclusions:**

A total of 116,915 repeats with a base length of at least three nucleotides were detected. The longest of these was a 54-base repeat with fourteen tandem copies. A significant number of repeats with a base length of 18, 24, 27 and 30 were detected, many with potentially novel proline-rich coding regions.

Detection of exact tandem repeats in the zebrafish genome leads to a wealth of information regarding potential polymorphic sites for VNTRs. The association of many of these repeats with potentially novel yet similar coding regions yields an exciting potential for disease associated genes. A web interface for querying repeats is available at http://bioinformatics.louisville.edu/zebrafish/. This portal allows for users to search for a repeats of a selected base size from any valid specified region within the 25 linkage groups.

## Background

### Zebrafish genome

Zebrafish are studied as model vertebrate organism with an early embryonic development similar to human development and genetics [1]. The zebrafish genome is approximately 1.7 billion bases long, spread out over 25 linkage groups [2], which is roughly half the size of the human genome. The rate of single nucleotide polymorphisms in the zebrafish genome is approximately one in 200 bases, compared to one per 1,000 bases in humans [3]. While this seems like a rare event, given that the zebrafish genome is 1.7 billion bases in length and the human genome is 3.2 billion bases long, that equates to eight million differences across the zebrafish genome and three million differences across the human genome. Several maps have been made for the zebrafish genome [4-7], including a low density map of 2,035 SNPs and 178 insertion/deletion (indel) events [3], a high density map of candidate SNPS [8], dinucleotide microsatellites [9], and mutant loci [10].

### Exact tandem repeats

Genomes can be thought of as an alphabet with four terminal symbols, or nucleotide bases, represented by A, C, G, T. The size of a genome can vary widely, ranging from a few thousand bases for viral genomes to tens of billions of bases for complex eukaryotic plants [11]. Across the genome of a particular species, a number of differences in the DNA sequences help to differentiate among individuals in the population.

Large regions of a genome may have undergone duplication events resulting in the repetition of the pattern in tandem, one right after another. For instance, if the pattern CAG is duplicated 10 times, it results in the sequence:

CAGCAGCAGCAGCAGCAGCAGCAGCAGCAG

This can be rewritten as (CAG)_10_, where CAG is the base pattern, and 10 is the number of times the pattern is repeated, also known as the copy number. In this example, CAG is a trinucleotide repeat, since the base sequence is three nucleotides long.

Tandemly repeated elements are prime locations for large scale polymorphisms within a population. As a result, microsatellite markers have been used to create high-density maps for a number of organisms, including the human [12], mouse [13] and rat [14].

In the human genome, at least 35 different human diseases have been associated with differences in repeat copy numbers [15]. Triplet repeats in particular are well studied, causing over 20 human neurological disorders and diseases associated with differences in the copy number of the repeat [15]. Expansion in the copy number of trinucleotide repeats expansion has been shown to play a major role in human disease, due to their disruption within the coding (exonic) and noncoding (intronic) regions of genes. Examples of diseases associated with the expansion of the trinucleotide CAG include Huntington's Disease, Smith's Disease, Kennedy's Disease, and Machado-Joseph Disease [16-18]. The repeat CAG within a coding region results in polyglutamine expansion, causing the protein to bind with GAPDH, thus affecting its ability to function in producing energy [19]. Other diseases, such as myotonic dystrophy, Fragile X syndrome, and Friedreich's ataxia [16,17] have been shown to be involved with trinucleotide expansions as well.

For some of these triplet repeat associated diseases, a small variation in the copy number can differentiate a healthy individual from an affected one. For instance, with Kennedy's Disease, a normal individual will have 21 tandem copies of a CAG repeat within an androgen receptor gene, while an individual with Kennedy's Disease will have 40-52 copies of the CAG repeat. Other triplet repeat associated diseases can result in a vast difference in the copy number. One instance of this is Fragile X, where a normal individual has 6-50 copies of a CCG repeat within the FMR1 gene and an affected individual may have 1,000 or more copies of CCG. In addition, dynamic mutations in the copy number of the tetramers CCTG [20] and TCAT [21] along with the pentamer ATTCT [22] and dodecamer CCCCGCCCCGCG [23] have been characterized within the human genome.

While the occurrence of short tandem repeats (STRs) of less than five base pairs within genomes have been well studied and used to create genetic maps [24-27] and looking for polymorphic markers within a population [28-30], less is known about the occurrence of polymorphic tandem repeats of longer lengths. Forensic tests use known polymorphic STRs to differentiate between different individuals [31]. In many cases, the pool of known polymorphic STRs is only a small subset existing within a species, leaving many STRs to be virtually unknown. Therefore, an efficient method for detecting regions susceptible to polymorphisms within a population is desired. Since STRs are prime candidates, a method for determining occurrences of short tandem repeats given a reference sequence is presented. Once the tandem repeats are discovered, the flanking regions can be mined for uniform PCR primers which can be used to experimentally test for variability in the copy number. Those regions which are then validated as having polymorphisms within the population can be used as regions for genetic markers.

The use of simple sequence repeats (SSRs) as markers for the construction of a genetic map within zebrafish has been previously proposed [32] by looking at a small subset of dinucleotide repeats. In the study by Goff et al., primer pairs were constructed for 25 different sequences, and 16 of the 17 pairs that produced products showed polymorphisms between two distantly related zebrafish lines, or a rate of 94%. Another study was able to map 2,000 microsatellite markers to the zebrafish genome by looking at simple sequence length polymorphisms (SSLPs) within CA dinucleotide repeats [9]. Now that a draft genome is available for the zebrafish, it is possible to look for tandem repeats in the genome beyond the traditional dinucleotide repeats.

### Tandem repeat/microsatellite programs

A number of programs have previously been written to detect repeats within genomes, including both interspersed repeats [33] and microsatellite repeats [34-36]. Databases of short period microsatellite repeats have been created for prokaryotic [37] and eukaryotic genomes [38-40]. However, none of these present the user with a method to biologically test whether or not these repeats have variable copy numbers in a population. The most popular biological approach to microsatellite DNA typing that has been used is PCR amplification. Therefore, a method to both detect regions of exact tandem repeats and PCR primers within the flanking sequence is proposed.

Methods to determine the location of markers such as the dinucleotide CA/TG repeats have been previously described by constructing PCR primers from comparative genomes which are then tested against the genome of interest. In addition, it is desired to detect whether or not polymorphic copy numbers exist for a particular locus, which can be accomplished by running the results of PCR amplification for a number of different individuals through an agarose gel.

### Project overview

This study detected exact tandem repeats of various length and copy number within the zebrafish genome. Once these regions were located, 500 bases flanking the beginning and ending of the repeat region were reported. These regions were additionally masked for known repeats using RepeatMasker. Primers were then identified in the flanking regions. These primers can then be used within PCR reactions to detect whether or not polymorphisms exist within a zebrafish population. Those regions where polymorphisms in the copy number exist can then be used to produce a higher resolution genetic map.

## Construction and content

### Sequence data

For the zebrafish genome sequencing project performed at the Sanger Centre, DNA was taken from ~1,000 zebrafish embryos [2]. The eighth assembly of the zebrafish genome (Zv8) dated 12-Dec-2008 was downloaded from Ensembl's FTP site [41] in the form of supercontigs. For the Zv8 assembly, there are 1,481,241,295 bases of finished sequence data, and 123,873,047 bases of unfinished sequences. The Zv8 assembly was derived from 9,816 clones assembled into 11,623 scaffolds as well as 6,882,050 reads from whole genome shotgun sequencing of a single Tuebingen, double haploid zebrafish.

### Repeat detection

Exact tandem repeats were detected using a method implemented as a perl script. Input into the script requires the sequence file in fasta format, the minimum and maximum repeat length to find, and a minimum repeat periodicity. Using these four inputs, tandem repeats can be detected for any sequence.

The algorithm proceeds across the length of the sequence, S, using a sliding window approach. The window size, w, is chosen according to the periodicity currently being considered. A substring S'_i,w _is taken from S, where i is the beginning location of the substring of length w. If S'_i,w _is equal to the previous substring found, then the repeat copy number is increased and the window proceeds to the next substring.

If S'_i,w _is not equal to the previous repeat, then it is tested to confirm that it is valid. In order to be a valid repeat, it is tested to ensure it does not contain a repeat of a smaller period. For instance, if w = 12, then S'_i,w _is tested to make sure that a repeat of length 1, 2, 3, 4, or 6 is not contained within it. As an example, if S'_i,w _= ACTAACTAACTA, then this would not be a valid repeat, since it contains the subrepeat (ACTA)_3_. All repeats found with a copy number greater than the user specified value are stored. Once all repeats have been detected, the repeats found are analyzed for overlaps due to frame shifting of the window. In the cases where overlaps are present, the repeat with the largest copy number is chosen as the repeat for this region.

After the repeats have been identified, the 500 bases (if available) upstream and downstream the repeat are reported. These regions will be used in the primer design step. At the conclusion of the program, information is printed concerning the base repeat, the copy number, beginning and ending location of the repeat, the whole repeat sequence, and the upstream and downstream regions.

This algorithm runs in C*n time, where C is the number of repeat lengths to search, and n is the length of the sequence. Since C is a constant generally computed beforehand, this factors to O(n) time complexity.

### Sequences used

In order to narrow down the search space, repeats with a length of at least three bases and a copy number of at least ten exact tandem repeats were detected. For instance, the repeat sequence AAGCT has a length of five; if AAGCT is found repeated at least 10 consecutive times (for instance, AAGCT AAGCT AAGCT AAGCT AAGCT AAGCT AAGCT AAGCT AAGCT AAGCT), then it is reported.

### PCR primers

After exact tandem repeat regions were detected, the 5' and 3' flanking regions were extracted and masked for low complexity and known repeat families using RepeatMasker [2] and MaskerAid [33]. PCR primers were detected within the flanking regions using MPrime 1.3 [42]. The resulting primer pairs can be used in PCR experiments to determine if polymorphisms in the copy number for this particular repeat occur in a given population. MPrime reports information concerning primer location, end annealing and self annealing scores, GC concentration, melting temperature, product size, and sequence similarity scores each of which is stored in a MySQL database for further use.

### Database structure

**Figure 1:**
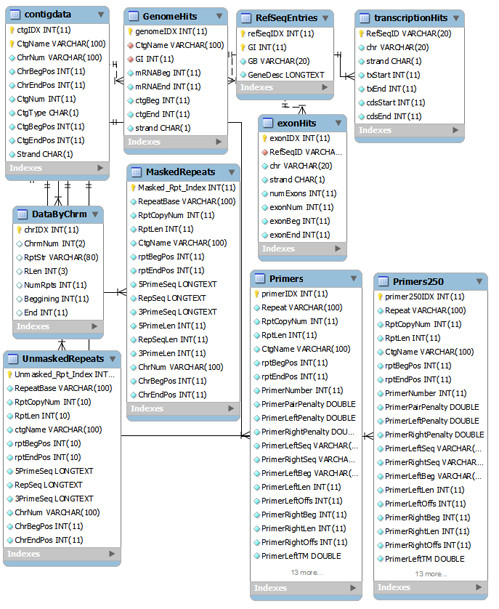
**Entity-relationship model for zebrafish repeats database**.

A relational database to store the zebrafish repeats was constructed using MySQL version 5.0.19. The database Zv8 is available with "select" privileges at the server http://kbrin.a-bldg.louisville.edu/ under the user "zebrafish" and password "daniorerio". Ten tables were created, primarily for genomic contig information, transcript location, and repeat identification. An entity-relationship (E-R) diagram of the database is provided in Fig. 1. A brief description of the content of each table follows.

**contigdata **contains information on assembled clones for the Zv8 assembly of the zebrafish genome, including their length and mapping location in relationship to a zebrafish linkage group. This information is taken directly from the Zv8_scaffold.agp file available from the Ensembl ftp site.

**RefSeqEntries **is a table of basic details of zebrafish RefSeq entries including RefSeqID, GenBank accession (GB), GenBank identifier (GI), and a brief description (GeneDesc). No sequence information for the RefSeq entries is stored locally within the Zv8 database since it can be readily accessed externally.

**transcriptionHits **contains coarse-level information about the transcription as well as translation start and stop of a RefSeq entry within a zebrafish linkage group. This information can then be used to quickly determine if any feature (such as a repeat) is genic or intergenic in nature.

**GenomeHits **contains the same information as transcriptionHits provided at a contig level as opposed to a linkage group level.

**exonHits **is a table providing exon-level information. This table contains information as far as the exon beginning and ending location and the exon number is concerned. exonHits can thus be used to determine if a feature is intronic or exonic in nature.

**UnmaskedRepeats **houses information for each detected repeat, including the contig in which it is found (ctgName), its location (rptBegPos, rptEndPos), its length and copy number (RptLen, RptCopyNum) and flanking sequence information (5PrimeSeq, 3PrimeSeq) which can be used to detect amplification primers.

**MaskedRepeats **contains the same information as UnmaskedRepeats with the exception that the flanking sequences have been masked for known repetitive elements and simple repeats using RepeatMasker as described in the previous section.

**DataByChrm **is a summary table taken from UnmaskedRepeats that can quickly be indexed when searching for repeats by linkage group.

**Primers **is a table containing information on amplification primers that have been detected using MPrime 1.3 in the flanking regions of the repeat masked entries of MaskedRepeats. Information contained in this table includes thermodynamic properties and parameters used in designing the primers.

**Primers250 **is a subset of Primers where the repeat region itself is 250 bases or fewer in length. The primers and corresponding repeats reported in this table are those most likely to be useful for studies of copy number polymorphisms, since they can be sequenced in a single run using traditional Sanger sequencing techniques.

## Utility

A list of the number of repeats found with base length three or more with a copy number of at least ten are given in Table 1. To no surprise, the number of tandem repeats is dominated by bases with a length of 3-, 4-, and 5-mers. The majority of these repeats are AT-rich, with the base repeat AAT and its reverse complement ATT accounting for 87% of all triplet tandem repeat loci (Additional file 1, Table S1). The repeats AGAT and its reverse complement ATCT compose 41% of all quadruplet tandem repeats (Additional file 1, Table S2) while 64% of all pentamer repeats are represented by the either the base sequence AATAT or its complement TTATA (Additional file 1, Table S3).

**Table 1:** Frequency of tandem repeats within the zebrafish genome Zv8 assembly by base length

**Repeat Len**	**#**	**Repeat Len**	**#**	**Repeat Len**	**#**
		
1-mers	N/A	19-mers	5	37-mers	3
2-mers	N/A	20-mers	6	38-mers	1
3-mers	37,383	21-mers	7	39-mers	2
4-mers	67,313	22-mers	9	40-mers	0
5-mers	11,767	23-mers	7	41-mers	2
6-mers	93	24-mers	18	42-mers	0
7-mers	1	25-mers	4	43-mers	1
8-mers	10	26-mers	7	44-mers	2
9-mers	1	27-mers	40	45-mers	2
10-mers	5	28-mers	5	46-mers	5
11-mers	5	29-mers	4	47-mers	0
12-mers	6	30-mers	117	48-mers	1
13-mers	3	31-mers	10	49-mers	1
14-mers	16	32-mers	5	50-mers	1
15-mers	6	33-mers	1	51-mers	1
16-mers	16	34-mers	2	52-mers	0
17-mers	5	35-mers	2	53-mers	1
18-mers	21	36-mers	9	54-mers	1

**Figure 2:**
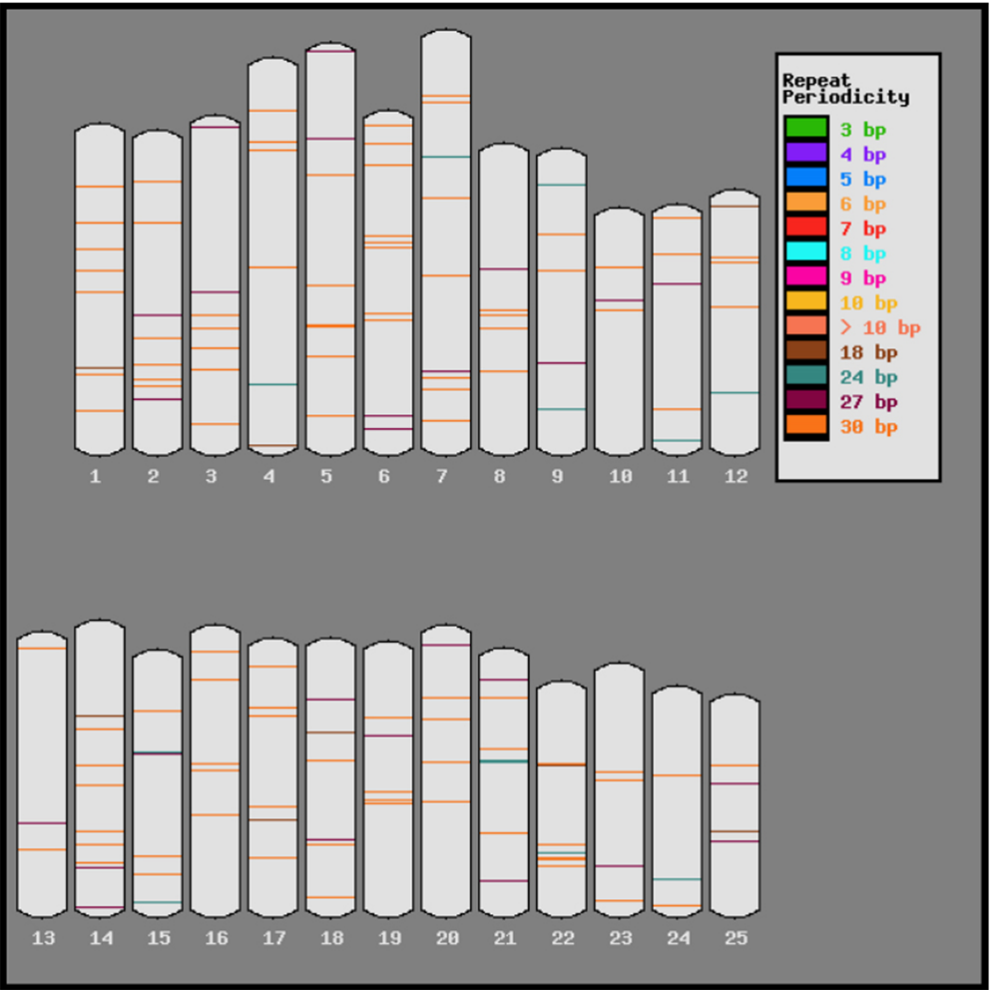
**Linkage group localization for repeats with a base length of 18, 24, 27 and 30**.

**Figure 3:**
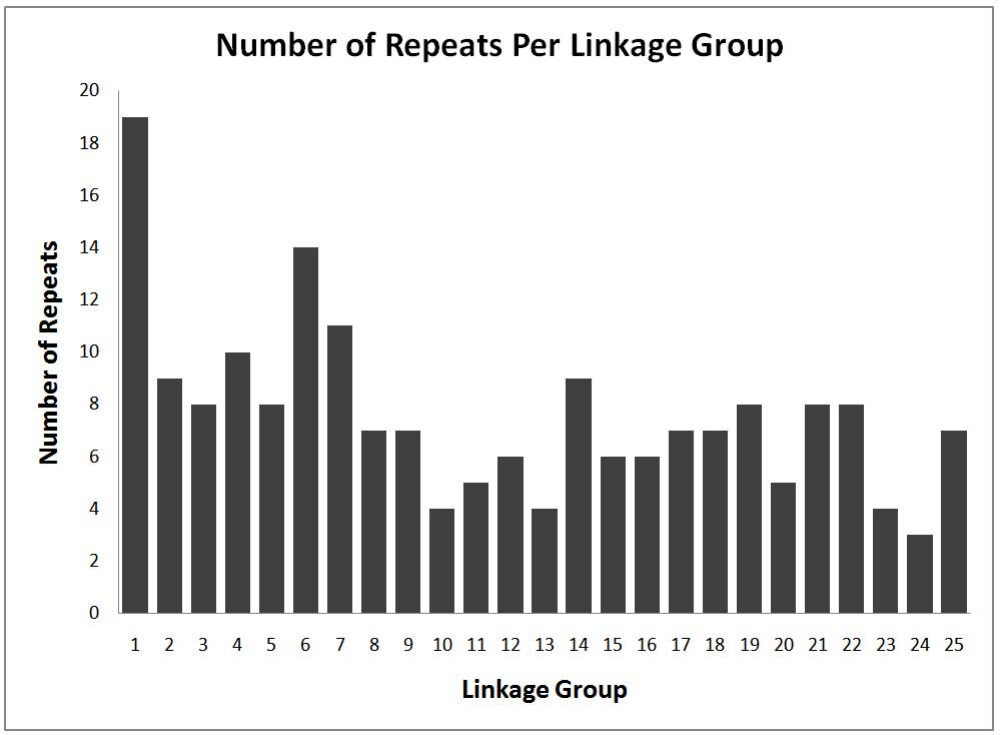
**Distribution of repeats of base length 18, 24, 27 and 30 among linkage groups**.

The results in Table 1 indicate a drastic drop off when the base repeat reaches a length of six. In addition, there appear to be spikes in the data when the base repeat length is a multiple of three, particularly with repeats with a base length of 18, 24, 27, and 30. Further analysis indicates that of the 191 detected exact tandem repeats of length 18, 24, 27, or 30, only 18 of these are within 10 kb of another such repeat. Further stringency finds that fourteen are within 500 bp, with six being direct overlaps due to a staggered repeat. Fig. 2 visually illustrates the linkage group localization of these repeats while Fig. 3 shows the distribution among linkage groups. A complete list of all 191 detected repeats is given in Additional file 2, Table S4 (length 18), Additional file 2, Table S5 (length 24), Additional file 2, Table S6 (length 27), and Additional file 2, Table S7 (length 30).

There is a single repeat with a base of 54 found within the Zv8 assembly. This is repeated fourteen times. This repeat is:

(CGAGTTCTTATCAGCTGTGTTGTCGCG

CGCGTACTGAATAGCGGTGTTGTCGCA)_14_

This repeat occurs in an intronic region of the gene with RefSeq ID NM_199842 (GenBank:41054140).

### Repeats with multiple of 3 base length

Repeats with a multiple of three base lengths are candidates for genic regions due to their ability to maintain the current reading frame while potentially providing for a repetitive amino acid sequence. Tandem repeats within a transcribed region are known to be affiliated with a plethora of genetic diseases. Such repeats can be classified into Type I (exonic) or Type II (intronic) expansions. The type of expansion characterizes not only the location of the repeats, but the underlying pathology as well [16].

Repeats occurring within transcribed regions were studied using the RefSeq gene sequence track from the December 2008 Goldenpath assembly of the zebrafish genome (corresponding to the Zv8 assembly) from the Table Browser of the UCSC genome browser [43]. A total of 131,383 exon locations were noted from 15,417 mapped loci. Each repeat location was compared against the RefSeq loci to determine if the repeat is close to a known gene (within 1,000 bp of the 5' or 3' end), overlaps a known gene region, or is contained within a known transcriptional unit. A total of 2,397 tandem repeats were found to be close to a known gene with nearly all of these (2,393) having a base repeat length of either three, four or five. These repeats are A+T rich. Forty-three tandem repeats were found to overlap intron/exon boundaries. Twenty-eight of these have a base length of three while eleven have a base length of four, three have a base length of five and one has a base length of 30. Forty-nine exonic tandem repeats were found, 30 with a base length of three, 15 with a base length of four, three with a base length of five, and one with a base length of 30 (Additional file 3, Table S8). Further analysis shows the majority of these repeats (35/49) are found in the 3' UTR. Two are found in the 5' UTR. The remaining twelve repeats are found within coding exons. There were 26,909 intronic tandem repeats detected. 7,624 of these intronic tandem repeats are of base length three, 15,410 are length four, while 2,752 are of base length five. For the 191 repeats with a base size of 18, 24, 27 or 30, forty-eight were found to occur within intronic regions; one within an exonic region; and one in an intron/exon boundary. The remaining 141 repeats appear to occur in intergenic regions.

Repeats with a base length of 18, 24, 27 or 30 were further characterized by looking at both the nucleic acid composition as well as the six possible protein translations. Out of the 21 repeats with a base of 18, eighteen can be translated into the consensus protein sequence PP[E|V]LPD (Table 2). This is particularly surprising since the majority are located in intergenic regions and are coded by six unique nucleotide sequences. For the repeats with a base length of 24, thirteen out of 18 can be translated into the consensus protein sequence PGPPPQLHA from one of five nucleotide sequences (Table 3). All but six of the repeats of base length 27 fall into one of three categories, translating into the consensus patterns V[L|M][IVNSK]S[C|G]VVAR, [L|P]A[L|Q]PAP[P|H]R[R|L|P], or L[P|A]E[W|R]PPPPE (Table 4). For the repeats of base length 30, one hundred and three can be translated into the perfectly conserved consensus pattern APAPERPPVS using one of seven different nucleotide sequences. One of these instances, in fact, is within a hypothetical protein sequence. An additional five instances translate into the peptide AP[A|V]QLPPVPP while eight translate to QLTRWPTPVL using one of two nucleotide patterns (Table 5).

**Table 2:** Repeats of length 18 with each of the six possible protein translations

GP	Repeat Base	Possible Protein Translations						#
1	ACCCTCCAGAGCTGCCAG	PPELPD	VWQLWR	PSRAAR	LQSCQT	GLAALE	GSSGGS	10
1	AGCTCTGGAGGGTCTGGC	PPELPD	VWQLWR	PSRAAR	LQSCQT	GLAALE	GSSGGS	3
1	ACTGGAGGGTCTGGCAGC	PPVLPD	VWQHWR	PSSAAR	LQCCQT	ALEGLA	GSTGGS	1
1	AGCTCTGGCGGGTCTGGC	PPELPD	VWQLWR	PARAAR	RQSCQT	GLAALA	SGSSGG	1
1	ACCCTCCAGTGCTGCCAG	PPVLPD	VHQHWR	PSSAAR	LQCCQT	GLAALE	SGSTGG	2
1	ACCCGCCAGAGCTGCCAG	PPELPD	VWQLWR	PARAAR	RQSCQT	GLAALA	GSSGGS	1
2	ACGCCGCAGCCAGAGTCG	CGVDSG	AASTLA	RRRLWL	ARVDAA	QPESTP	SQSRRR	1
3	ATCGTGGCCCCCTCGTCC	RPSWPP	VHRGPL	SIVAPS	RGPRWT	RGGHDG	EGATMD	1
4	CCCTGTGGTGCTGTGTGT	CGAVCP	VVLCVP	WCCVSL	GTHSTT	QGHTAP	RDTQHH	1

GP: grouping; #: number of instances found in the Zv8 assembly. Note the repeat base has been reordered by lexicographical order.

**Table 3:** Repeats of length 24 with each of the six possible protein translations

GP	Repeat Base	Possible Protein Translations						#
1	ACGCTCCAGGCCCTCCGCAGCTCC	PGPPQLHA	AWSCGGPG	SRPSAAPR	QALRSSTL	ERGAAEGL	SVELRRAW	6
1	AGCGTGGAGCTGCGGAGGGCCTGG	PGPPQLHA	AWSCGGPG	SRPSAAPR	QALRSSTL	ERGAAEGL	SVELRRAW	4
1	ACGCCCCAGGCCCTCCGCAGCTCC	PGPPQLHA	AWSCGGPG	PRPSAAPR	QALRSSTP	GRGAAEGL	GVELRRAW	1
1	AGCTGCGGAGGGCCTGGGGCGTGG	PGPPQLHA	AWSCGGPG	PRPSAAPR	QALRSSTP	LRGAAELG	GVELRRAW	1
1	ACGCTCCAGGCCCTCGGCAGCTGC	PGPRQLHA	TLQALGSC	SRPSAAAR	GACSCRGP	ERAAAELG	SVQLPRAW	1
2	AAGCCCGAGGCGACGCCATTGGAG	GLLQWRRL	EATPLEKP	RRRHWRSP	GDAIGEAR	SGFSNGVA	RASPMASP	1
2	AAGGCCGAGGCGACGCCATTGGAG	GLLQWRRL	EATPLEKA	RRRHWRRP	GDAIGEGR	SAFSNGVA	MASPRPSP	1
3	AAGCGGATTTTTGACGCGCGAGTG	*SGFLTRE	EADF*RAS	KRIFDARV	LARQKSAS	HSRVKNPL	TRASKIRF	1
4	AAGCGCCGGTGAGCCCTCGCCCTC	ALEGEGSP	RLRARAHR	A*GRGLTG	R*ALALKR	AGEPSPSS	PVSPRPQA	1
5	AAGCTCAGGCGGCGGCCATTCAGG	GGGHSGSS	AAAIQEAQ	RRPFRKLR	*AS*MAAA	PELPEWPP	LSFLNGRR	1

GP: grouping; #: number of instances found in the Zv8 assembly. Note the repeat base has been reordered by lexicographical order.

**Table 4:** Repeats of length 27 with each of the six possible protein translations

GP	Repeat Base	All Possible Protein Translations						#
1	AACACAGCTGATAAGAACTCGCGCGAC	VLISCVVAR	RATTQLIRT	SRDNTADKN	FLSAVLSRE	SSYQLCCRA	LARQHS**E	2
1	AACACAGCTGATCAGTACGCGCGCGAC	VLISCVVAR	RATTQLIST	ARDNTADQY	Y*SAVLSRA	RTDQLCCRA	RARQHS*SV	1
1	AGCTGTGTTGTCGCGCGCGTTATGATC	VMISCVVAR	RATTQLIIT	ARDNTADHN	L*SAVLSRA	RYDQLCCRA	RARQHS*S*	2
1	AGCTGTGTTGTCGCGCGCGTTCTGATC	VLISCVVAR	RATTQLIRT	ARDNTADQN	F*SAVLSRA	RSDQLCCRA	RARQHS*SE	1
1	AATAGCGGTGTTGTCGCGCGCGTTCTG	VLNSGVVAR	RATTPLFRT	ARDNTAIQN	F*IAVLSRA	RSE*RCCRA	RARQHRYSE	1
1	AACACCGCTCTTCAGTACGCGCGCGAC	VLKSGVVAR	RATTPLFST	ARDNTALQY	Y*RAVLSRA	RTEERCCRA	RARQHRSSV	1
1	AGCTGTGTTGTCGCGCGAGTTCTTATC	VLISCVVAR	RATTQLIRT	SRDNTADKN	FLSAVLSRE	SSYQLCCRA	LARQHS**E	3
1	AACACAGCTGATAAGAACGCGCGCGAC	VLISCVVAR	RATTQLIRT	ARDNTADKN	FLSAVLSRA	RSYQLCCRA	RARQHS**E	1
1	AACACCGCTACTCAGTACGCGCGCGAC	VLSSGVVAR	RATTPLLST	ARDNTATQY	Y*VAVLSRA	RTE*RCCRA	RARQHRYSV	1
1	AACACAGCTGATCAGAACGCGCGCGAC	VLISCVVAR	RATTQLIRT	ARDNTADQN	VLSRAF*SA	RSDQLCCRA	RARQHS*SE	1
1	AACACAGCTGACAAGAACTCGCGCGAC	VLVSCVVAR	RATTQLTRT	SRDNTADKN	FLSAVLSRE	SSCQLCCRA	LARQHS*QE	1
2	ACCCAGGCTCCTCGCCCTGCCGGCGCC	LALPAPPRL	SPCRRHPGS	RPAGATQAP	RSLGGAGRA	GAWVAPAGR	EPGWRRQGE	1
2	ACCCAGACGTCTCGCCCTGCCGGCGCC	LALPAPPRR	SPCRRHPDV	RPAGATQTS	RRLGGAGRA	DVWVAPAGR	TSGWRRQGE	1
2	ACGTCTGGGTGGCGCCGGCAGGGCGAG	LALPAPPRR	SPCRRHPDV	RPAGATQTS	RRLGGAGRA	DVWVAPAGR	TSGWRRQGE	1
2	ACGTCTGGGTGGCGCCGGCTGGGCGAG	LAQPAPPRR	SPSRRHPDV	RPAGATQTS	RRLGGAGWA	DVWVAPAGR	TSGWRRLGE	1
2	ACAGGCCTCCAGCCCAGCCGGCTCCCC	PAQPAPHRP	GSRLGWRPV	SPAGSPQAS	GGLWGAGWA	LGWRPVGSR	PAGLEACGE	1
3	AATGGCCGCCGCCTCCTGAGCTTCCTG	LPEWPPPPE	SSGGGGHSG	LRRRRPFRK	S*MAAAS*A	AQEAAAIQE	FLNGRRLLS	1
3	AAGCTCAGGAGGCGGCGGCCATTCAGG	LPEWPPPPE	SSGGGGHSG	LRRRRPFRK	S*MAAAS*A	AQEAAAIQE	FLNGRRLLS	2
3	AGCTCAGGCGGCGGCGGCCATTCAGGG	LPEWPPPPE	SSGGGGHSG	LRRRRPFRE	P*MAAAA*A	AQAAAAIQG	SLNGRRRLS	1
3	AGCGAGCTCGGGAGGCGGCGGCCATTC	LAEWPPPPE	SSGGGGHSA	LGRRRPFSE	R*MAAASRA	AREAAAIQR	SLNGRRLPS	1
3	AATGGCCGCCGCCGCCTGAGCTTCCTG	LPEWPPPPE	SSGGGGHSG	LRRRRPFRK	S*MAAAA*A	AQAAAAIQE	FLNGRRRLS	3
3	AAGCTCAGGAGGCGGCGGCCGTTCAGG	LPERPPPPE	SSGGGGRSG	LRRRRPFRK	S*TAAAS*A	AQEAAAVQE	FLNGRRLLS	3
3	AATGGCCGCCGCCGCCTGAGCTCCCTG	LPEWPPPPE	SSGGGGHSG	LRRRRPFRE	P*MAAAA*A	AQAAAAIQG	LNGRRRLSS	1
3	AACGGCCGCCGCCTCCTGAACTCCCTG	LPERPPPPE	SSGGGGRSG	FRRRRPFRE	P*TAAAS*T	VQEAAAVQG	LNGRRLLNS	2
4	AAGACCAGAGGGGAGCCGGCGGGGCTG	G*RPEGSRR	AGGAEDQRG	LKTRGEPAG	PRRLPSGLQ	PAGSPLVFS	PPAPLWSSA	1
4	CCCCTCTGGTCTCCTGCCCTGCCGGCT	GRRPEGSRQ	AGRAGDQRG	QETRGEPAG	PCRLPSGLL	PAGSPLVSC	LPAPLWSPA	1
5	AACTCTATTGAGTGTCAGGTCATCTCC	SSPTLLSVR	HLQLY*VSG	ISNSIECQV	DLTNRVGD	T*HSIELEM	PDTQ*SWR*	1
6	AAAGCAACAACTCCACCAACATCAGCT	QLHQHQLKQ	NSTNIS*SN	TPPTSAKAT	CCFS*CWWS	VALADVGGV	LL*LMLVEL	1
7	AACAGCAGAGCCATTCACTGACCCCAG	H*PQNSRAI	TDPRTAEPF	LTPEQQSHS	*MALLFWGQ	EWLCCSGVS	NGSAVLGSV	1
8	AAGGAGCCGGGCTGGGGCCGGCAGGGC	AGRARSRAG	PAGQGAGLG	RQGKEPGWG	APARLLALP	PQPGSLPCR	PSPAPCPAG	1

GP: grouping; #: number of instances found in the Zv8 assembly. Note the repeat base has been reordered by lexicographical order.

**Table 5:** Repeats of length 30 with each of the six possible protein translations

GP	Repeat Base	All possible protein translations						#
1	AGCCCCTGAGCGCCCTCCAGTGTCGGCTCC	APAPERPPVS	DTGGRSGAGA	RHWRALRGWS	RLQPLSALQC	GSSP*APSSV	TLEGAQGLEP	32
1	AGAGCGCCCGCCAGTGTCGGCTCCAGCCCC	APAPERPPVS	DTGGRSGAGA	RHWRALWGWS	RLQPQSARQC	GSSPRAPASV	TLAGALGLEP	12
1	ACACTGGAGGGCGCTCAGGGGCTGGAGCCG	APAPERPPVS	DTGGRSGAGA	RHWRALRGWS	RLQPLSALQC	GSSP*APSSV	TLEGAQGLEP	16
1	ACACTGGCGGGCGCTCTGGGGCTGGAGCCG	APAPERPPVS	DTGGRSGAGA	RHWRALWGWS	RLQPQSARQC	GSSPRAPASV	TLAGALGLEP	12
1	ACACTGGAGGGCGCTCTGGGGCTGGAGCCG	APAPERPPVS	DTGGRSGAGA	RHWRALWGWS	RLQPQSALQC	GSSPRAPSSV	TLEGALGLEP	15
1	AGAGCGCCCTCCAGTGTCGGCTCCAGCCCC	APAPERPPVS	DTGGRSGAGA	RHWRALWGWS	RLQPQSALQC	GSSPRAPSSV	TLEGALGLEP	14
1	ACACTGGCGGGCGCTCGGGGGCTGGAGCCG	APAPERPPVS	DTGGRSGAGA	RHWRALGGWS	RLQPPSARQC	GSSPRAPASV	TLAGARGLEP	2
2	ACTGGAGGCAGCTGGACTGGAGCCGGCGGG	APVQLPPVPP	AGGTGGSWTG	PAGLEAAGLE	RRDWRQLDWS	LQSSCLQSRR	SSPAASSPAG	2
2	ACAGGAGGCAGCTGGGCTGGAGCCGGCGGG	APAQLPPVPP	AGGTGGSWAG	PAGQEAAGLE	RRDRRQLGWS	LQPSCLLSRR	SSPAASCPAG	1
2	AGCTGCCTCCAGTCCCGCCGGCTCCTGCCC	APAQLPPVPP	AGGTGGSWAG	PAGLEAAGQE	RRDWRQLGRS	LLPSCLQSRR	SSPAGSCPAA	1
2	ACTGGAGGCAGCTGGGCAGGAGCCGGCGGG	APAQLPPVPP	AGGTGGSWAG	PAGLEAAGQE	RRDWRQLGRS	LLPSCLQSRR	SSPAGSCPAA	1
3	AAGATGGCCGACTCCAGTCCTCCAGCTCAC	QLTRWPTPVL	SSQDGRLQSS	AHKMADSSP	WRTGVGHLVS	GGLESAIL*A	EDWSRPSCEL	7
3	ACTGGAGTCGGCCATCTTGTGAGCTGGAGG	QLTRWPTPVL	SSQDGRLQSS	AHKMADSSP	WRTGVGHLVS	GGLESAIL*A	EDWSRPSCEL	1
4	AAACTGCCGCAAGGCTCCAAATACTTCTCC	KLPQGSKYFS	NCRKAPNTSP	TAARLQILLQ	LEKYLEPCGS	WRSIWSLAAV	GEVFGALRQF	1

GP: grouping; #: number of instances found in the Zv8 assembly. Note the repeat base has been reordered by lexicographical order.

### Repeat distribution

**Figure 4:**
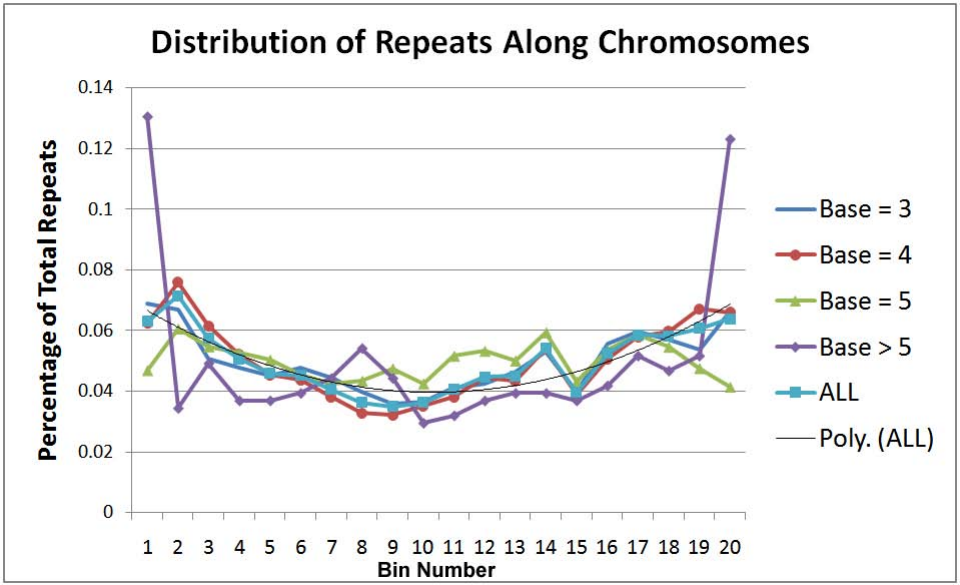
**Distribution of repeats across linkage groups**. The bin number in the x-axis represents increments of 5% across each linkage group, while the y-axis represents the percentage of all repeats falling within the respective bin.

The distribution of repeats along each of the linkage groups was examined. Each linkage group was divided into twenty segments along their length with each segment representing 5% of the total length of the linkage group. Repeats were placed in a bin based upon their begin location. Repeat base lengths of 3 bp, 4 bp, 5 bp, and >5 bp were examined. A chi-square test was performed for each base length and linkage group combination independently (results not shown). For 19 degrees of freedom, the critical χ^2 ^value for rejecting the null hypothesis of a uniform distribution with p = 0.95 is 30.14 and for p = 0.99, the value is 36.19. For triplet repeats, the null hypothesis is rejected for all 25 linkage groups with the highest differences occurring in bins 1 and 20, representing the telomeric regions of the linkage group. The null hypothesis is rejected for all 25 linkage groups for 4 base repeats with bins 1, 3, 18 and 20 showing the largest differences. Pentamer repeats also exhibit non-uniform distribution as well since the null hypothesis is rejected for all linkage groups. Repeats with a base length greater than 5 do not seem to have as many statistically significant hot spots since the null hypothesis is only rejected for eleven linkage groups at a level of p = 0.95. However, the bins showing the largest difference are bins 18 and 20 in the telomeric region at one end of the linkage group. These results are summarized in Fig. 4 which shows the distribution of repeats along the chromosome. As Fig. 4 indicates, there seems to be a strong preference for repeats in the telomeric regions of the linkage group. In fact, as the trend line indicates, the further away from the center of the linkage group, the more likely it is that a repeat will occur.

## Discussion

### Comparison to other eukaryotic genomes

The most comprehensive resource for microsattelite repeats is the Tandem Repeats Database [44]. Our analysis of the exact tandem repeats in the zebrafish genome were compared to the human genome (hg19 build), mouse (mm8), rat (rn4), fugu (fr1), and tetraodon (tetNig1) genomes available in the tandem repeats database (Table 6). Based on these queries, it is shown that the detected exact tandem repeats within the zebrafish genome are much more prevalent, occurring at a rate of once out of every 12,659 based compared to the closest genome, mouse, which shows a frequency of 1/45,498 bases. For each of the genomes (with the exception of tetraodon), the most prevalent repeat base length is 4, with roughly twice as many tandem repeats as triplet base lengths. One interesting observation is that the number of hexamer repeats is highly variable and is found at a low frequency in the zebrafish. A comparison of the repeats of length 18, 24, 27, and 30 suggest the zebrafish is somewhat unique. It must be noted that this analysis should be taken with care, since the other genomes are older assemblies and the repeat detection mechanisms between Tandem Repeats Finder and our approach while similar, are not identical.

**Table 6:** Comparison of exact tandem repeats within various eukaryotic genomes

			Base Size							
Genome	Build	Genome Size (MB)	All	3	4	5	6	18, 24, 27, 30	Long	Frequency
zebrafish	Zv8	1480	116,915	37,383	67,313	11,767	93	191	54	1/12,659
mouse	Mm8	2600	57,145	16,022	33,430	5,186	2,066	17	68	1/45,498
rat	Rn4	2800	41,422	16,746	22,077	1,213	930	35	70	1/67,597
fugu	Fr1	395	3,785	997	2,375	366	33	0	48	1/104,359
tetraodon	TetNig1	350	1,808	1,287	419	82	14	0	12	1/193,584

Long represents the longest base size repeat detected. Frequency calculated as the genome size divided by the number of total repeats.

### Conserved repeat regions

While there is not any direct evidence that more than one of the exact repeats of length 18, 24, 27 or 30 are part of a coding region, the striking conservation at the protein level cannot be ignored, particularly since the nucleotide sequence in not necessarily conserved. Looking at the consensus peptide translations, in can be seen that many of these are proline-rich. Most of these contain the pattern PxxP, which has been shown to bind to SH3 domain containing proteins [45]. This indicates the potential of novel signaling and protein-interaction genes [46-48].

NCBI blastp analysis of the nr database searching for the repeat APAPERPPVS yields six hypothetical zebrafish proteins from the RefSeq XP division (GenBank:18954121, GenBank:189515106, GenBank:189514738, GenBank:12581872, GenBank:189532449, GenBank:125844672, GenBank:189518247) detected using GNOMON [49]. Most of the blastp hits yield hypothetical proteins. Annotated hits include a serum response binding protein with the repeat RPAVERPAVE (GI 89271263); inclusion membrane protein with the repeat APAPEAPAPE (GenBank:169667289); cell wall type 2 protein with the repeat KPPVEKPPVY (GenBank:2347094); and an outer membrane adhesion-like protein with the repeat APAPEPSPAP (GenBank:170774881). These annotations further underline the potential for these repeats to belong to novel proline-rich genes. Further research into these repeats is needed to prove conclusive biological evidence.

Since the zebrafish genome is currently not at a finished state, it must be noted that some of the tandem repeats detected may result from misassemblies. In addition, the repeat detection program as currently constructed only detects exact tandem repeats. If a repeat region is long enough, a stutter in the repeat base will result in two adjacent repeat regions being reported. It is highly likely that some repetitive regions will go undetected or will be presented as truncated using this method. The repeats detected and presented using this approach will not be a complete set of repeat regions but should yield a high percentage of all tandemly repeating elements with a repeat copy number of ten or more.

Since the tiling path for the Zv8 reference genome is a consensus from over 1,000 different animals, the consensus sequence may under- or over-represent the repetitive tandemly repeating regions within an individual. Further insight might be gathered by looking at individual trace files which could indicate repeats with variable copy numbers. However, gaps in the Zv8 tiling path are filled using a single animal that has been sequenced using whole genome sequencing methods. In addition, Zv8 provides 6.5-7× coverage, indicating that only commonly found variations would be detected within the trace files.

## Conclusions

In this research, we detected exact tandem repeats within the zebrafish genome with a base length of at least three and a copy number of at least 10 with the end goal of detecting regions likely to be vulnerable to VNTRs. A total of 116,915 such regions were detected, along with primer pairs in the flanking regions that can be used for biological assays. The high incidence of repeats with a base length of 18, 21, 24, 27, and 30 suggests that many of these are involved in genic regions. While none of these are within known protein coding sequences, the high similarity of potential amino acid translations suggests novel coding sequences containing highly repetitive regions. Our results should serve as a resource for zebrafish molecular biologists interested in studying potential diseases associated with these repeats, as well as serve as a source for construction of a higher resolution genetic map than is currently available.

## Availability and requirements

A web interface for querying repeats is available at http://bioinformatics.louisville.edu. This portal allows for users to search for a repeats of a selected base size from any valid specified region within the 25 linkage groups. The information returned is a graphical representation of the repeats as well as a link to a tab-delimited text file containing information concerning the linkage group, chromosomal repeat begin and end positions, repeat base size, repeat base sequence, copy number, and the highest scoring forward and reverse primer from the flanking sequence. In addition, the repeats are available for download as custom tracks for the UCSC [43] and FishMap [50] genome browsers. The MySQL Zv8 database can be accessed directly with select privledges from the MySQL server http://kbrin.a-bldg.louisville.edu with the username 'zebrafish' and the password 'daniorerio'.

## Authors' contributions

ER was responsible for all aspects of this work, including project design, database design, analysis, and manuscript preparation. All authors read and approved of the final manuscript.

## Supplementary Material

Additional file 1 **Tables containing information on the number of trinucleotide, quadruplet and pentamer tandem repeats detected in the zebrafish genome Zv8 assembly**. Click here for file

Additional file 2 **Tables containing information on the repeat instances with a base length size of 18, 24, 27 and 30 detected in the zebrafish genome Zv8 assembly**. Click here for file

Additional file 3 **Table containing information on the exact tandem repeat instances found within coding regions of RefSeq entries mapped in the zebrafish genome Zv8 assembly**. Click here for file
