# Supplementary material for: Database of exact tandem repeats in the Zebrafish genome
Source: BMC Genomics. 2010 Jun 1;11:347. doi: 10.1186/1471-2164-11-347 (PMC2901318; doi:10.1186/1471-2164-11-347)
Supplement: Additional file 1 — Tables containing information on the number of trinucleotide, quadruplet and pentamer tandem repeats detected in the zebrafish genome Zv8 assembly. [file 1471-2164-11-347-S1.DOC]

**Table S1: Frequency of trinucleotide tandem repeats in the zebrafish genome Zv8 assembly.**

| **Repeat** | **Number of Instances** |  | **Repeat** | **Number of Instances** |  | **Repeat** | **Number of Instances** |
| --- | --- | --- | --- | --- | --- | --- | --- |
| **ATT/TAT/TTA** | 16,491 |  | **ATG/GAT/TGA** | 508 |  | **CTG/GCT/TGC** | 17 |
| **AAT/ATA/TAA** | 16,178 |  | **ATC/CAT/TCA** | 499 |  | **ACC/CAC/CCA** | 11 |
| **AGT/GTA/TAG** | 799 |  | **CTT/TCT/TTC** | 343 |  | **AGC/CAG/GCA** | 7 |
| **ACT/CTA/TAC** | 796 |  | **AAG/AGA/GAA** | 325 |  | **GGT/GTG/TGG** | 4 |
| **GTT/TGT/TTG** | 695 |  | **AGG/GAG/GGA** | 27 |  | **CGT/GTC/TCG** | 2 |
| **AAC/ACA/CAA** | 659 |  | **CCT/CTC/TCC** | 21 |  | **CCG/CGC/GCC** | 1 |

**Table S2: Frequency of quadruplet tandem repeats in the zebrafish genome Zv8 assembly.**

| **Repeat** | **Number of Instances** |  | **Repeat** | **Number of Instances** |  | **Repeat** | **Number of Instances** |
| --- | --- | --- | --- | --- | --- | --- | --- |
| **ATCT** | 13,830 |  | **AACT** | 461 |  | **AACG** | 40 |
| **AGAT** | 13,610 |  | **AGTT** | 433 |  | **CCTG** | 35 |
| **ATCC** | 4,429 |  | **ACGG** | 331 |  | **CGTT** | 27 |
| **ATGG** | 4,379 |  | **CCGT** | 328 |  | **AAGT** | 24 |
| **AAAT** | 3,400 |  | **AACC** | 321 |  | **ACTT** | 24 |
| **ATTT** | 3,292 |  | **GGTT** | 301 |  | **CGGT** | 21 |
| **ACAG** | 2,832 |  | **ACTC** | 272 |  | **ACCG** | 12 |
| **CTGT** | 2,762 |  | **AGTG** | 258 |  | **ATCG** | 10 |
| **AATG** | 2,441 |  | **ACGC** | 245 |  | **AGCC** | 8 |
| **ATTC** | 2,302 |  | **CGTG** | 217 |  | **CTGG** | 7 |
| **CTTT** | 1,935 |  | **ACTG** | 206 |  | **AAGC** | 6 |
| **AAAG** | 1,776 |  | **AGTC** | 172 |  | **CTTG** | 5 |
| **AATC** | 1,557 |  | **CCTT** | 157 |  | **ATGC** | 4 |
| **ATTG** | 1,324 |  | **AAGG** | 139 |  | **AGGG** | 3 |
| **ACAT** | 993 |  | **AATT** | 92 |  | **CCCT** | 3 |
| **ATGT** | 924 |  | **ACCT** | 88 |  | **ACCC** | 2 |
| **AAAC** | 611 |  | **AGGT** | 75 |  | **GGGT** | 1 |
| **GTTT** | 543 |  | **AGGC** | 45 |  |  |  |

**Table S3: Frequency of pentamer tandem repeats within the zebrafish Zv8 assembly.**

| **Repeat** | **Number** |  | **Repeat** | **Number** |  | **Repeat** | **Number** |  | **Repeat** | **Number** |
| --- | --- | --- | --- | --- | --- | --- | --- | --- | --- | --- |
| **AATAT** | 3771 |  | **AATAC** | 21 |  | **AAATG** | 5 |  | **CCTGG** | 2 |
| **ATATT** | 3729 |  | **AATCT** | 21 |  | **AACAG** | 5 |  | **CTGTT** | 2 |
| **AAATT** | 723 |  | **CTTTT** | 21 |  | **AAGGC** | 5 |  | **AAGGG** | 1 |
| **AATTT** | 648 |  | **ACTTT** | 18 |  | **ATCTC** | 5 |  | **ACACG** | 1 |
| **ATTTT** | 592 |  | **AGATT** | 16 |  | **ATTTC** | 5 |  | **ACATC** | 1 |
| **AAAAT** | 552 |  | **AGGAT** | 16 |  | **ATTTG** | 5 |  | **ACATG** | 1 |
| **AATAG** | 273 |  | **ATCCT** | 16 |  | **ACAGG** | 4 |  | **ACCTG** | 1 |
| **ATTCT** | 238 |  | **AAAGT** | 15 |  | **ACTCT** | 4 |  | **ACCAT** | 1 |
| **AAAAC** | 96 |  | **AGTTC** | 12 |  | **ACTTG** | 4 |  | **ACGTT** | 1 |
| **AAACT** | 86 |  | **GTGTT** | 12 |  | **AGGCC** | 4 |  | **AGATG** | 1 |
| **AGTAT** | 82 |  | **AAAGG** | 9 |  | **CCTTG** | 4 |  | **AGCCT** | 1 |
| **ACTAT** | 76 |  | **ATCTT** | 9 |  | **AATGT** | 3 |  | **AGCTC** | 1 |
| **GTTTT** | 75 |  | **AAAGC** | 8 |  | **ACACT** | 3 |  | **AGTCC** | 1 |
| **AGTTT** | 69 |  | **AACTG** | 8 |  | **AAACC** | 2 |  | **AGTGT** | 1 |
| **AACAT** | 56 |  | **CCTTT** | 8 |  | **AACCT** | 2 |  | **ATGGT** | 1 |
| **AAGAG** | 55 |  | **CTTTG** | 8 |  | **AACGT** | 2 |  | **ATGTG** | 1 |
| **CTCTT** | 52 |  | **AAATC** | 7 |  | **AACTC** | 2 |  | **ATTGG** | 1 |
| **ATGTT** | 43 |  | **AACAC** | 7 |  | **AAGCC** | 2 |  | **CCCTG** | 1 |
| **AAAAG** | 36 |  | **AAGAT** | 7 |  | **AAGGT** | 2 |  | **CCTCT** | 1 |
| **AATTG** | 34 |  | **AAGTC** | 7 |  | **AATGC** | 2 |  | **CGTTT** | 1 |
| **ATATG** | 30 |  | **AGAGT** | 7 |  | **ACATT** | 2 |  | **CTCTG** | 1 |
| **AATTC** | 28 |  | **AACTT** | 6 |  | **ACTGG** | 2 |  | **CTGGG** | 1 |
| **ATATC** | 27 |  | **AAGTT** | 6 |  | **AGAGG** | 2 |  | **CTTGG** | 1 |
| **ATTGT** | 22 |  | **CTTGT** | 6 |  | **AGGTT** | 2 |  |  |  |
