# Supplementary material for: Database of exact tandem repeats in the Zebrafish genome
Source: BMC Genomics. 2010 Jun 1;11:347. doi: 10.1186/1471-2164-11-347 (PMC2901318; doi:10.1186/1471-2164-11-347)
Supplement: Additional file 2 — Tables containing information on the repeat instances with a base length size of 18, 24, 27 and 30 detected in the zebrafish genome Zv8 assembly. [file 1471-2164-11-347-S2.DOC]

**Table S4: Repeat instances with a base size of 18.**

| **CHR** | **BEG** | **END** | **BASE**  **SIZE** | **BASE**  **SEQUENCE** | **COPY**  **NUMBER** | **REPEAT**  **TYPE** |
| --- | --- | --- | --- | --- | --- | --- |
| 1 | 27626 | 27841 | 18 | TGCCAGACCCTCCAGAGC | 12 |  |
| 1 | 10509248 | 10509463 | 18 | CCTCCAGTGCTGCCAGAC | 12 |  |
| 1 | 44346152 | 44346331 | 18 | CCAGAGCTGCCAGACCCG | 10 | I NM_001159372 |
| 4 | 16186673 | 16186924 | 18 | GCTGCCAGACCCTCCAGA | 14 | I NM_131697 |
| 4 | 71074485 | 71074754 | 18 | CGTCCATCGTGGCCCCCT | 15 |  |
| 6 | 55728758 | 55728937 | 18 | CTGGAGGGTCTGGCAGCT | 10 |  |
| 7 | 62751873 | 62752052 | 18 | GGCAGCTCTGGAGGGTCT | 10 |  |
| 8 | 41178771 | 41178986 | 18 | CCAGAGCTGCCAGACCCT | 12 | I NM_213167 |
| 9 | 47427370 | 47427567 | 18 | GCTGCCAGACCCTCCAGA | 11 |  |
| 10 | 15936240 | 15936581 | 18 | CCTCCAGAGCTGCCAGAC | 19 |  |
| 12 | 1842836 | 1843015 | 18 | GGTCTGGCAGCTCTGGCG | 10 |  |
| 14 | 16720204 | 16720401 | 18 | CTGGAGGGTCTGGCAGCA | 11 |  |
| 17 | 32610339 | 32610590 | 18 | CGCAGCCAGAGTCGACGC | 14 |  |
| 18 | 16222700 | 16222897 | 18 | TGTGGTGCTGTGTGTCCC | 11 |  |
| 19 | 16134494 | 16134799 | 18 | CTCCAGAGCTGCCAGACC | 17 |  |
| 19 | 16134495 | 16134800 | 18 | TCCAGAGCTGCCAGACCC | 17 |  |
| 21 | 19856410 | 19856625 | 18 | CCAGAGCTGCCAGACCCT | 12 |  |
| 21 | 19884087 | 19884284 | 18 | GCTGCCAGACCCTCCAGT | 11 |  |
| 22 | 14616857 | 14617054 | 18 | GCTGCCAGACCCTCCAGA | 11 |  |
| 25 | 24154363 | 24154758 | 18 | AGCTCTGGAGGGTCTGGC | 22 |  |
| 25 | 26125132 | 26125419 | 18 | GCTGCCAGACCCTCCAGA | 16 |  |

**Table S5: Repeats instances with a base size of 24.**

| **CHR** | **BEG** | **END** | **BASE**  **SIZE** | **BASE SEQUENCE** | **COPY**  **NUMBER** | **REPEAT**  **TYPE** |
| --- | --- | --- | --- | --- | --- | --- |
| 1 | 10510231 | 10510686 | 24 | GCAGCTCCACGCTCCAGGCCCTCC | 19 |  |
| 1 | 30330381 | 30330644 | 24 | CCTCAAGCGCCGGTGAGCCCTCGC | 11 |  |
| 4 | 59941606 | 59941941 | 24 | GAGGCGACGCCATTGGAGAAGCCC | 14 |  |
| 4 | 59941940 | 59942539 | 24 | CCGAGGCGACGCCATTGGAGAAGG | 25 |  |
| 6 | 55727680 | 55727919 | 24 | TGGAGCTGCGGAGGGCCTGGAGCG | 10 |  |
| 7 | 22552439 | 22552774 | 24 | GGCGGCGGCCATTCAGGAAGCTCA | 14 |  |
| 7 | 63886501 | 63886740 | 24 | CTGGAGCGTGGAGCTGCGGAGGGC | 10 |  |
| 9 | 5647475 | 5647714 | 24 | CTGGAGCGTGGAGCTGCGGAGGGC | 10 |  |
| 9 | 47428164 | 47428715 | 24 | TCCACGCTCCAGGCCCTCCGCAGC | 23 |  |
| 11 | 42760067 | 42760306 | 24 | CCCAGGCCCTCCGCAGCTCCACGC | 10 |  |
| 12 | 36583474 | 36583809 | 24 | TCCACGCTCCAGGCCCTCCGCAGC | 14 |  |
| 15 | 17851547 | 17851810 | 24 | GCTCCAGGCCCTCCGCAGCTCCAC | 11 |  |
| 15 | 45957844 | 45958083 | 24 | GAGTGAAGCGGATTTTTGACGCGC | 10 | I NM_205646 |
| 21 | 19857476 | 19857715 | 24 | TCCACGCTCCAGGCCCTCCGCAGC | 10 |  |
| 21 | 19884809 | 19885072 | 24 | TCCACGCTCCAGGCCCTCCGCAGC | 11 |  |
| 22 | 30854636 | 30855019 | 24 | GGGCCTGGAGCGTGGAGCTGCGGA | 16 |  |
| 24 | 34763183 | 34763422 | 24 | GCGTGGAGCTGCGGAGGGCCTGGG | 10 | I NM_001033748 |

**Table S6: Repeats instances with a base size of 27.**

| **CHR** | **BEG** | **END** | **BASE SIZE** | **BASE**  **SEQUENCE** | **COPY**  **NUMBER** | **REPEAT**  **TYPE** |
| --- | --- | --- | --- | --- | --- | --- |
| 1 | 28110 | 28379 | 27 | GCCGCCGCCTGAGCTTCCTGAATGGCC | 10 |  |
| 2 | 33258931 | 33259227 | 27 | AGTACGCGCGCGACAACACAGCTGATC | 11 | I NM_194383 |
| 2 | 49024720 | 49025043 | 27 | GTGTTGTCGCGCGCGTTCTGAATAGCG | 12 |  |
| 3 | 945589 | 945858 | 27 | CAACTCCACCAACATCAGCTAAAGCAA | 10 |  |
| 3 | 31819249 | 31819545 | 27 | GTGTTGTCGCGCGCGTTCTGATCAGCT | 11 | I NM_198911 |
| 3 | 36127900 | 36128331 | 27 | GCCTGAGCTCCCTGAATGGCCGCCGCC | 16 |  |
| 4 | 16187337 | 16187687 | 27 | CCTGAACGGCCGCCGCCTCCTGAACTC | 13 | I NM_131697 |
| 4 | 16187702 | 16188079 | 27 | GCCTCCTGAACTCCCTGAACGGCCGCC | 14 | I NM_131697 |
| 5 | 581375 | 581671 | 27 | CACTGACCCCAGAACAGCAGAGCCATT | 11 | I NM_001045284 |
| 5 | 16683143 | 16683412 | 27 | GTGTTGTCGCGCGAGTTCTTATCAGCT | 10 |  |
| 6 | 55728136 | 55728459 | 27 | GGCGGCGGCCGTTCAGGAAGCTCAGGA | 12 |  |
| 6 | 58056154 | 58056558 | 27 | CGCGCGACAACACCGCTACTCAGTACG | 15 | I NM_001109724 |
| 6 | 58056735 | 58057382 | 27 | GCTGATAAGAACGCGCGCGACAACACA | 24 | I NM_001109724 |
| 7 | 62751447 | 62751716 | 27 | AGCTCAGGCGGCGGCGGCCATTCAGGG | 10 |  |
| 8 | 22096337 | 22096606 | 27 | AGGCGGCGGCCATTCAGGAAGCTCAGG | 10 |  |
| 9 | 21636604 | 21636900 | 27 | CTGAAGACCAGAGGGGAGCCGGCGGGG | 11 | I NM_001102644,  I NM_001102631 |
| 9 | 38788914 | 38789264 | 27 | TCAGGAGGCGGCGGCCGTTCAGGAAGC | 13 | I NM_001171585 |
| 10 | 15936961 | 15937284 | 27 | CTGAATGGCCGCCGCCGCCTGAGCTTC | 12 | I NM_001122654 |
| 11 | 13380698 | 13381021 | 27 | GACAACACAGCTGATAAGAACTCGCGC | 12 |  |
| 12 | 20445444 | 20445713 | 27 | GGGCGAGACGTCTGGGTGGCGCCGGCA | 10 |  |
| 13 | 34683503 | 34684285 | 27 | GTGTTGTCGCGCGCGTTATGATCAGCT | 29 |  |
| 13 | 34733748 | 34734530 | 27 | GTGTTGTCGCGCGCGTTATGATCAGCT | 29 |  |
| 14 | 44874234 | 44874746 | 27 | GAGCTCGGGAGGCGGCGGCCATTCAGC | 19 |  |
| 14 | 52227912 | 52228262 | 27 | TCATCTCCAACTCTATTGAGTGTCAGG | 13 |  |
| 15 | 18279000 | 18279350 | 27 | AGCTCAGGAGGCGGCGGCCGTTCAGGA | 13 |  |
| 16 | 24808970 | 24809320 | 27 | GCCGGCAGGGCAAGGAGCCGGGCTGGG | 13 | I NM_001044848 |
| 17 | 13248414 | 13248683 | 27 | GCCCTGCCGGCTCCCCTCTGGTCTCCT | 10 |  |
| 18 | 10143064 | 10143468 | 27 | CGCGCGACAACACAGCTGATAAGAACT | 15 |  |
| 18 | 36066029 | 36066379 | 27 | AGGCGGCGGCCATTCAGGAAGCTCAGG | 13 |  |
| 18 | 37137706 | 37138407 | 27 | CTCCAGCCCAGCCGGCTCCCCACAGGC | 26 |  |
| 19 | 16135242 | 16135592 | 27 | GCCTCCTGAGCTTCCTGAATGGCCGCC | 13 |  |
| 20 | 2466415 | 2467467 | 27 | TGATCAGAACGCGCGCGACAACACAGC | 39 | I NM_001003990 |
| 21 | 4512020 | 4512478 | 27 | TCAGTACGCGCGCGACAACACCGCTCT | 17 |  |
| 21 | 42241340 | 42241879 | 27 | GTGTTGTCGCGCGAGTTCTTATCAGCT | 20 |  |
| 23 | 36424867 | 36425217 | 27 | AGTTCTTATCAGCTGTGTTGTCGCGCG | 13 |  |
| 25 | 11710663 | 11710986 | 27 | GCCGGCTGGGCGAGACGTCTGGGTGGC | 12 |  |
| 25 | 15241564 | 15241833 | 27 | GCTCCTCGCCCTGCCGGCGCCACCCAG | 10 |  |
| 25 | 26125754 | 26126050 | 27 | CCTGAGCTTCCTGAATGGCCGCCGCCG | 11 |  |

**Table S7: Repeats instances with a base size of 30.**

| **CHR** | **BEG** | **END** | **BASE**  **SIZE** | **BASE SEQUENCE** | **COPY**  **NUMBER** | **REPEAT**  **TYPE** |
| --- | --- | --- | --- | --- | --- | --- |
| 1 | 86705 | 87154 | 30 | CTCCAGCCCCAGAGCGCCCGCCAGTGTCGG | 15 |  |
| 1 | 155427 | 155756 | 30 | CAGTGTCGGCTCCAGCCCCAGAGCGCCCTC | 11 |  |
| 1 | 854878 | 855507 | 30 | GGCGCTCAGGGGCTGGAGCCGACACTGGAG | 21 |  |
| 1 | 10508312 | 10508941 | 30 | CCAGCTCACAAGATGGCCGACTCCAGTCCT | 21 |  |
| 1 | 17257306 | 17258055 | 30 | CAGTGTCGGCTCCAGCCCCTGAGCGCCCTC | 25 |  |
| 1 | 22149617 | 22149976 | 30 | GCTCCAGCCCCTGAGCGCCCTCCAGTGTCG | 12 |  |
| 1 | 26094060 | 26094569 | 30 | GGAGCCGACACTGGAGGGCGCTCTGGGGCT | 17 |  |
| 1 | 30121387 | 30121716 | 30 | GCGCTCTGGGGCTGGAGCCGACACTGGCGG | 11 |  |
| 1 | 45572953 | 45573342 | 30 | CTGGAGCCGACACTGGAGGGCGCTCTGGGG | 13 |  |
| 1 | 52521693 | 52521992 | 30 | GGAGCCGGCGGGACTGGAGGCAGCTGGACT | 10 | I NM_001145574 |
| 1 | 52522467 | 52522796 | 30 | GGAGCCGACACTGGCGGGCGCTCTGGGGCT | 11 | I NM_001145574 |
| 1 | 52524321 | 52524620 | 30 | GGAGCCGGCGGGACTGGAGGCAGCTGGACT | 10 | I NM_001145574 |
| 1 | 52525095 | 52525424 | 30 | GGAGCCGACACTGGCGGGCGCTCTGGGGCT | 11 | I NM_001145574 |
| 2 | 8359916 | 8360275 | 30 | GGCGCTCTGGGGCTGGAGCCGACACTGGCG | 12 | I NM_001100084 |
| 2 | 16048004 | 16048333 | 30 | GCTCTGGGGCTGGAGCCGACACTGGCGGGC | 11 | I NM_001126393 |
| 2 | 37398565 | 37398984 | 30 | GCTCCAGCCCCAGAGCGCCCGCCAGTGTCG | 14 |  |
| 2 | 42623698 | 42624207 | 30 | GCTCCAGCCCCTGAGCGCCCTCCAGTGTCG | 17 | I NM_205674 |
| 2 | 42624209 | 42624538 | 30 | CTCCAGCCCCTGAGCGCCCTCCAGTGTCGG | 11 | I NM_205674 |
| 2 | 45278495 | 45279094 | 30 | GGGCTGGAGCCGACACTGGAGGGCGCTCTG | 20 |  |
| 2 | 46474718 | 46475047 | 30 | GCGCTCTGGGGCTGGAGCCGACACTGGAGG | 11 |  |
| 3 | 36127274 | 36127663 | 30 | CCAGCTCACAAGATGGCCGACTCCAGTCCT | 13 |  |
| 3 | 38699736 | 38700125 | 30 | GGCTCCAGCCCCTGAGCGCCCTCCAGTGTC | 13 |  |
| 3 | 42391893 | 42392282 | 30 | CAGTGTCGGCTCCAGCCCCAGAGCGCCCTC | 13 |  |
| 3 | 46383202 | 46383591 | 30 | GAGCGCCCTCCAGTGTCGGCTCCAGCCCCT | 13 |  |
| 3 | 56358800 | 56359159 | 30 | GAGCGCCCTCCAGTGTCGGCTCCAGCCCCT | 12 |  |
| 4 | 8797639 | 8797938 | 30 | GCGCTCTGGGGCTGGAGCCGACACTGGAGG | 10 |  |
| 4 | 14616920 | 14617519 | 30 | CCGACACTGGAGGGCGCTCAGGGGCTGGAG | 20 |  |
| 4 | 16186017 | 16186316 | 30 | CACAAGATGGCCGACTCCAGTCCTCCAGCT | 10 | I NM_131697 |
| 4 | 37846424 | 37846843 | 30 | GCTCCAGCCCCTGAGCGCCCTCCAGTGTCG | 14 | I NM_001114585 |
| 5 | 23532463 | 23532792 | 30 | CAGCCCCTGAGCGCCCTCCAGTGTCGGCTC | 11 |  |
| 5 | 44081047 | 44081346 | 30 | GCTCCAGCCCCTGAGCGCCCTCCAGTGTCG | 10 | I/E NM_001083014 |
| 5 | 51521064 | 51521453 | 30 | CAGCCCCTGAGCGCCCTCCAGTGTCGGCTC | 13 |  |
| 5 | 51760162 | 51760461 | 30 | GCTCGGGGGCTGGAGCCGACACTGGCGGGC | 10 |  |
| 5 | 57485576 | 57486325 | 30 | CAGTGTCGGCTCCAGCCCCAGAGCGCCCTC | 25 |  |
| 5 | 68495880 | 68496299 | 30 | GCTCCAGCCCCTGAGCGCCCTCCAGTGTCG | 14 |  |
| 6 | 1574251 | 1574670 | 30 | CTGCCGCAAGGCTCCAAATACTTCTCCAAA | 14 | I NM_001110010 |
| 6 | 4997408 | 4997737 | 30 | CCCTGAGCGCCCTCCAGTGTCGGCTCCAGC | 11 |  |
| 6 | 4997429 | 4997728 | 30 | GGCTCCAGCCCCTGAGCGCCCTCCAGTGTC | 10 |  |
| 6 | 8949786 | 8950085 | 30 | CCCAGAGCGCCCGCCAGTGTCGGCTCCAGC | 10 |  |
| 6 | 22040442 | 22041341 | 30 | GGGCGCTCAGGGGCTGGAGCCGACACTGGA | 30 |  |
| 6 | 23248494 | 23248853 | 30 | GGGGCTGGAGCCGACACTGGCGGGCGCTCG | 12 |  |
| 6 | 24330722 | 24331201 | 30 | TGTCGGCTCCAGCCCCAGAGCGCCCGCCAG | 16 |  |
| 6 | 36672161 | 36673750 | 30 | GAGCCGACACTGGAGGGCGCTCAGGGGCTG | 53 |  |
| 6 | 37774900 | 37775289 | 30 | AGTCCTCCAGCTCACAAGATGGCCGACTCC | 13 |  |
| 7 | 11370650 | 11372149 | 30 | GCTCCAGCCCCTGAGCGCCCTCCAGTGTCG | 50 |  |
| 7 | 12485663 | 12485992 | 30 | GGGCGCTCAGGGGCTGGAGCCGACACTGGA | 11 | I NM_001004581,  I NM_200799 |
| 7 | 30386866 | 30387795 | 30 | GGCGCTCTGGGGCTGGAGCCGACACTGGAG | 31 |  |
| 7 | 44822468 | 44822827 | 30 | CGCCCTCCAGTGTCGGCTCCAGCCCCTGAG | 12 |  |
| 7 | 63887847 | 63888206 | 30 | GCCATCTTGTGAGCTGGAGGACTGGAGTCG | 12 |  |
| 7 | 65877940 | 65878719 | 30 | GCTCCAGCCCCTGAGCGCCCTCCAGTGTCG | 26 |  |
| 7 | 71830194 | 71830823 | 30 | GGGCGCTCAGGGGCTGGAGCCGACACTGGA | 21 |  |
| 8 | 29822239 | 29822628 | 30 | GGGGCTGGAGCCGACACTGGAGGGCGCTCT | 13 | I NM_001004123 |
| 8 | 30761969 | 30762448 | 30 | GGCGCTCTGGGGCTGGAGCCGACACTGGAG | 16 |  |
| 8 | 33303929 | 33304588 | 30 | CGGCTCCAGCCCCAGAGCGCCCTCCAGTGT | 22 | I NM_001020514 |
| 8 | 41158413 | 41158712 | 30 | GGGCTGGAGCCGACACTGGAGGGCGCTCTG | 10 | I NM_213167 |
| 8 | 41178216 | 41178575 | 30 | AGTCCTCCAGCTCACAAGATGGCCGACTCC | 12 | I NM_213167 |
| 9 | 14895181 | 14895540 | 30 | CCAGCCCCAGAGCGCCCTCCAGTGTCGGCT | 12 |  |
| 9 | 21637769 | 21638068 | 30 | GCGCTCTGGGGCTGGAGCCGACACTGGAGG | 10 | I NM_001102644,  I NM_001102631 |
| 10 | 9836821 | 9841290 | 30 | GCCCCAGAGCGCCCGCCAGTGTCGGCTCCA | 149 |  |
| 10 | 17895350 | 17896009 | 30 | CCCAGAGCGCCCGCCAGTGTCGGCTCCAGC | 22 |  |
| 11 | 1136885 | 1137334 | 30 | GCGCTCTGGGGCTGGAGCCGACACTGGCGG | 15 |  |
| 11 | 8023545 | 8024234 | 30 | GAGCGCCCTCCAGTGTCGGCTCCAGCCCCT | 23 |  |
| 11 | 37028284 | 37028613 | 30 | CAGTGTCGGCTCCAGCCCCAGAGCGCCCTC | 11 |  |
| 12 | 11264274 | 11264843 | 30 | CCCTCCAGTGTCGGCTCCAGCCCCTGAGCG | 19 |  |
| 12 | 12327768 | 12328067 | 30 | GCTCAGGGGCTGGAGCCGACACTGGAGGGC | 10 |  |
| 12 | 20446422 | 20446811 | 30 | GGCGCTCAGGGGCTGGAGCCGACACTGGAG | 13 |  |
| 13 | 2012424 | 2012843 | 30 | CGCTCAGGGGCTGGAGCCGACACTGGAGGG | 14 |  |

**Table S7 (continued).**

| **CHR** | **BEG** | **END** | **BASE**  **SIZE** | **BASE SEQUENCE** | **COPY**  **NUMBER** | **REPEAT**  **TYPE** |
| --- | --- | --- | --- | --- | --- | --- |
| 13 | 39619016 | 39619495 | 30 | CGGCTCCAGCCCCAGAGCGCCCTCCAGTGT | 16 |  |
| 14 | 19064903 | 19065322 | 30 | TGGGGCTGGAGCCGACACTGGCGGGCGCTC | 14 | I NM_001114729 |
| 14 | 25880066 | 25880485 | 30 | GAGCGCCCTCCAGTGTCGGCTCCAGCCCCT | 14 | I NM_131736 |
| 14 | 29561538 | 29562017 | 30 | GCGCTCTGGGGCTGGAGCCGACACTGGCGG | 16 | E NM_200688 |
| 14 | 38131138 | 38131737 | 30 | CAGCCCCAGAGCGCCCTCCAGTGTCGGCTC | 20 | I NM_001020465 |
| 14 | 40696023 | 40697582 | 30 | GGAGCCGACACTGGAGGGCGCTCAGGGGCT | 52 |  |
| 14 | 44138475 | 44138834 | 30 | GCCGGCGGGACAGGAGGCAGCTGGGCTGGA | 12 |  |
| 15 | 10022535 | 10023044 | 30 | GGGGCTGGAGCCGACACTGGAGGGCGCTCA | 17 |  |
| 15 | 37089186 | 37091435 | 30 | GGGCTGGAGCCGACACTGGAGGGCGCTCTG | 75 |  |
| 15 | 40636335 | 40636814 | 30 | CTCTGGGGCTGGAGCCGACACTGGCGGGCG | 16 |  |
| 16 | 3638135 | 3638494 | 30 | GCTCCAGCCCCTGAGCGCCCTCCAGTGTCG | 12 |  |
| 16 | 9066616 | 9066915 | 30 | GGGGCTGGAGCCGACACTGGAGGGCGCTCT | 10 |  |
| 16 | 24809550 | 24809879 | 30 | GGCGCTCAGGGGCTGGAGCCGACACTGGAG | 11 | I NM_001044848 |
| 16 | 25896437 | 25896736 | 30 | CAGCTCACAAGATGGCCGACTCCAGTCCTC | 10 |  |
| 16 | 34345973 | 34346902 | 30 | CCGACACTGGAGGGCGCTCAGGGGCTGGAG | 31 |  |
| 17 | 4096736 | 4097035 | 30 | GCTCCAGCCCCTGAGCGCCCTCCAGTGTCG | 10 | I NM_001020772 |
| 17 | 11830410 | 11831039 | 30 | GCTCCAGCCCCTGAGCGCCCTCCAGTGTCG | 21 |  |
| 17 | 13247302 | 13247871 | 30 | CAGTGTCGGCTCCAGCCCCAGAGCGCCCTC | 19 |  |
| 17 | 30346066 | 30346605 | 30 | GCTCCAGCCCCTGAGCGCCCTCCAGTGTCG | 18 |  |
| 17 | 39807138 | 39807587 | 30 | CGGCTCCAGCCCCTGAGCGCCCTCCAGTGT | 15 |  |
| 18 | 21346204 | 21346653 | 30 | CTCCAGCCCCAGAGCGCCCGCCAGTGTCGG | 15 | I NM_152968 |
| 18 | 37136584 | 37137213 | 30 | CAGTGTCGGCTCCAGCCCCAGAGCGCCCTC | 21 |  |
| 18 | 46901586 | 46901945 | 30 | GGGCTGGAGCCGACACTGGAGGGCGCTCTG | 12 |  |
| 19 | 12816020 | 12816529 | 30 | CAGCCCCTGAGCGCCCTCCAGTGTCGGCTC | 17 |  |
| 19 | 26762603 | 26763322 | 30 | CCCAGAGCGCCCGCCAGTGTCGGCTCCAGC | 24 |  |
| 19 | 26764071 | 26764370 | 30 | TCCAGTCCCGCCGGCTCCTGCCCAGCTGCC | 10 |  |
| 19 | 28175259 | 28175588 | 30 | GGAGCCGGCGGGACTGGAGGCAGCTGGGCA | 11 |  |
| 19 | 28846791 | 28847210 | 30 | CTCCAGTGTCGGCTCCAGCCCCAGAGCGCC | 14 |  |
| 20 | 12321742 | 12322041 | 30 | CCAGTGTCGGCTCCAGCCCCAGAGCGCCCG | 10 |  |
| 20 | 16422397 | 16422696 | 30 | CTCCAGCTCACAAGATGGCCGACTCCAGTC | 10 |  |
| 20 | 24463062 | 24463361 | 30 | CAGTGTCGGCTCCAGCCCCAGAGCGCCCTC | 10 |  |
| 20 | 31861832 | 31862131 | 30 | GCGCTCTGGGGCTGGAGCCGACACTGGAGG | 10 |  |
| 21 | 8089760 | 8090089 | 30 | CCCTCCAGTGTCGGCTCCAGCCCCAGAGCG | 11 |  |
| 21 | 17479711 | 17480190 | 30 | GCTCCAGCCCCTGAGCGCCCTCCAGTGTCG | 16 |  |
| 21 | 33250900 | 33251289 | 30 | GGGGCTGGAGCCGACACTGGAGGGCGCTCT | 13 | I NM_001080580 |
| 22 | 14279984 | 14280283 | 30 | GCTCCAGCCCCTGAGCGCCCTCCAGTGTCG | 10 |  |
| 22 | 14280288 | 14280617 | 30 | CAGCCCCTGAGCGCCCTCCAGTGTCGGCTC | 11 |  |
| 22 | 29394981 | 29395340 | 30 | CGCTCAGGGGCTGGAGCCGACACTGGAGGG | 12 |  |
| 22 | 31601613 | 31602092 | 30 | AGCCCCAGAGCGCCCGCCAGTGTCGGCTCC | 16 |  |
| 22 | 31938519 | 31938908 | 30 | CCCTCCAGTGTCGGCTCCAGCCCCTGAGCG | 13 |  |
| 22 | 33376451 | 33376780 | 30 | CCCAGAGCGCCCGCCAGTGTCGGCTCCAGC | 11 |  |
| 23 | 19041237 | 19041716 | 30 | GGGGCTGGAGCCGACACTGGCGGGCGCTCT | 16 |  |
| 23 | 20509483 | 20509872 | 30 | GCCCTCCAGTGTCGGCTCCAGCCCCTGAGC | 13 |  |
| 23 | 42911605 | 42912054 | 30 | CCGCCAGTGTCGGCTCCAGCCCCAGAGCGC | 15 |  |
| 24 | 15413222 | 15413611 | 30 | GCGCTCTGGGGCTGGAGCCGACACTGGCGG | 13 | I NM_200379 |
| 24 | 39522240 | 39522569 | 30 | CTGGGGCTGGAGCCGACACTGGCGGGCGCT | 11 | I NM_212882 |
| 25 | 11712129 | 11712668 | 30 | GGAGCCGACACTGGAGGGCGCTCAGGGGCT | 18 |  |
| 25 | 11725707 | 11726246 | 30 | GGAGCCGACACTGGAGGGCGCTCAGGGGCT | 18 | I NM_001122750 |
