# Supplementary material for: Database of exact tandem repeats in the Zebrafish genome
Source: BMC Genomics. 2010 Jun 1;11:347. doi: 10.1186/1471-2164-11-347 (PMC2901318; doi:10.1186/1471-2164-11-347)
Supplement: Additional file 3 — Table containing information on the exact tandem repeat instances found within coding regions of RefSeq entries mapped in the zebrafish genome Zv8 assembly. [file 1471-2164-11-347-S3.DOC]

**Table S8: Exonic repeats.**

| **CHR** | **RefSeq ID** | **Gene Symbol** | **Orientation** | **Nucleotide Repeat^1^** | **Effect** |
| --- | --- | --- | --- | --- | --- |
| 1 | NM_001030080 | Zgc:109892 | - | TAT | 3' UTR |
| 1 | NM_001145576 | Zgc:194962 | - | TATTA | 3' UTR |
| 1 | NM_199812 | Zgc:66472 | - | TCA | D |
| 1 | NM_199812 | Zgc:66472 | - | TCA | D |
| 2 | NM_001142672 | Insra | - | ATC | 3' UTR |
| 2 | NM_212893 | Dpyd | + | GATT | 3' UTR |
| 2 | NM_212723 | Mknk2a | + | TTGT | 3' UTR |
| 3 | NM_001111082 | Ifnphi2 | + | TATT | 3' UTR |
| 4 | NM_001013273 | Caprin2 | - | AAT | S |
| 6 | NM_001127473 | zgc:175146 | + | CAT | 3' UTR |
| 5 | NM_001123312 | myst3 | + | CAA | Q |
| 5 | NM_001083828 | Aatf | + | ATG | D |
| 5 | NM_001103124 | Zgc:171734 | - | TAA | 3' UTR |
| 6 | NM_001080600 | Shq1 | - | CAT | D |
| 6 | NM_001113376 | Lepr | - | TCT | E |
| 6 | NM_207099 | Zgc:77222 | + | ATT | 3' UTR |
| 7 | NM_001003452 | Tipin | + | TGA | D |
| 8 | NM_131722 | Mapk14a | + | GTGC | 3' UTR |
| 9 | NM_212827 | Mao | - | CTAT | 3' UTR |
| 9 | NM_213216 | Arglu1a | + | TTCTC | 3' UTR |
| 9 | NM_001004496 | Ofd1 | + | ATC | S |
| 10 | NM_001077716 | Zgc:153126 | - | GTG | 5' UTR |
| 11 | NM_205635 | Slc35b2 | - | CACG | 3' UTR |
| 11 | NM_001098195 | Zgc:162897 | - | AATAT | 3' UTR |
| 13 | NM_201500 | Elovl61 | - | AAT | 3' UTR |
| 14 | NM_199516 | St3gal5 | - | TCT | 5' UTR |
| 14 | NM_131421 | Nkx2.5 | + | TTCT | 3' UTR |
| 14 | NM_200688 | Zgc:64189 | - | GCGCTCTGGGGCTGGAGCCGACACTGGCGG | APAPERPPVS |
| 15 | NM_001083025^2^ | Pcsk7 | + | GTGC | 3' UTR |
| 15 | NM_001083025^2^ | Pcsk7 | - | CACG | 3' UTR |
| 16 | NM_001005923 | Stmn2a | - | GATA | 3' UTR |
| 16 | NM_131160 | Pou47 | + | TTA | 3' UTR |
| 16 | NM_131160 | Pou47 | + | TTA | 3' UTR |
| 16 | NM_001017694 | Osr2 | + | TGA | 3' UTR |
| 18 | NM_200643 | Slc30a4 | - | CAT | 3' UTR |
| 19 | NM_214753 | Gpd1 | - | CAT | 3' UTR |
| 20 | NM_001128330 | Ppil4 | - | ATG | 3' UTR |
| 20 | NM_001030169 | Sippa1l1 | - | TCC | G |
| 21 | NM_200911 | zgc:56064 | + | GTGA | 3' UTR |
| 21 | NM_001080586 | zgc:158319 | - | GCAC | 3' UTR |
| 21 | NM_001080058 | Zgc:158291 | + | ATT | 3' UTR |
| 21 | NM_001037678 | Zgc:123236 | - | TGT | Q |
| 21 | NM_212900 | Zgc:77058 | - | AAG | 3' UTR |
| 22 | NM_200728 | Zgc:73143 | + | ATG | 3' UTR |
| 22 | NM_001161337 | Dpp4 | + | ATT | 3' UTR |
| 22 | NM_213118 | Sox2 | - | TAA | 3' UTR |
| 25 | NM_131262^3^ | Myod1 | - | AAAG | 3' UTR |
| 25 | NM_131262^3^ | Myod1 | - | AAAG | 3' UTR |
| 25 | NM_131262^3^ | Myod1 | - | GAAA | 3' UTR |

^1^Repeats are reported with respect to the genomic strand. For genes occurring on the '-' strand, the actual repeats should be reverse complemented. ^2^The sequence NM_001083025 maps to multiple locations in the genome according to the UCSC goldenpath RefSeq track information. ^3^The three AAAG repeats for NM_131262 are in close proximity and thus should be considered as a single repeat unit.
